# Fecal Sample Collection Method for Wild Birds-Associated Microbiome Research: Perspectives for Wildlife Studies

**DOI:** 10.3390/ani10081349

**Published:** 2020-08-04

**Authors:** Luca Borrelli, Adriano Minichino, Antonino Pace, Ludovico Dipineto, Alessandro Fioretti

**Affiliations:** Department of Veterinary Medicine and Animal Productions, Università degli Studi di Napoli Federico II, via Delpino 1, 80137 Naples, Italy; adrianominichino@gmail.com (A.M.); antonino.pace@unina.it (A.P.); ludovico.dipineto@unina.it (L.D.); fioretti@unina.it (A.F.)

**Keywords:** noninvasive method, collecting box, birds feces, microbiome, wildlife

## Abstract

**Simple Summary:**

This paper describes an easy-to-build box for the noninvasive collection of feces from wild birds or small wild animals (up to 1 kg), including a plastic storage box, a plastic tray, and a vinyl-coated hardware cloth. This method could minimize potential contamination and allow for cross-study comparisons on gut microbiomes for wildlife medicine, conservation, ecology, and evolutionary biology.

**Abstract:**

Gut microbial communities play important roles in host health, modulating development, nutrient acquisition, immune and metabolic regulation, behavior and diseases. Wildlife microbiome studies and host–microbe interaction and exploration might be an important goal for evolutionary biology, conservation, and ecology. Therefore, collection and sampling methods must be considered before choosing a microbiome-based research plan. Since the fecal microbial community reflects the true gut community better than that of cloacal swab samples and only few nondestructive methods have been described, we propose an easy-to-build box for a noninvasive fecal collection method. The main components of the collection box include a plastic storage box, a plastic tray, a vinyl-coated hardware cloth, and a 10% bleach solution. In the plastic box, the tray is positioned under the raised grate, where the bird is placed, to reduce the risk of contamination of the fecal samples. This procedure could simplify handling and processing phases in wild birds or other animals. It might represent a cheap and useful method for research studies, wildlife rescue center activities, veterinary practices, and conservation practitioners.

## 1. Introduction

Host-associated microbiomes are of fundamental importance for vertebrates deeply influencing host health and diseases through their impacts on the immune system, digestion, development, and behavior [1,2]. Much like humans, animals are colonized by bacteria, which partition their bodies into distinct habitats (e.g., skin, hair or fur, feathers integument, oral, reproductive and gut mucosa) [3,4,5]. The microbiome affects host fitness, population demography, adaptability, conservation, and management of species. Microbiome studies can also increase our understanding of non-native species invasion, host physiology and health response to pathogens and chemical contamination, anthropogenic environmental disturbances, and host ability to tolerate climate change that can reduce gut microbial alpha-diversity in some wild animal populations [6]. Despite the ample potential for microbiome research to shape host evolution and ecology for animal health and conservation, wildlife microbiome studies are still a developing field [7,8]. Currently, although human microbiome research protocols are well-defined, there are no standardized sets of best methodology guiding the collection of microbiome samples from wildlife. Gut microflora is typically sampled either by fecal collection (from environmental substrate or placing materials in a container), rectal or cloacal swabbing, or by destructively sampling (from dead animals) the intestinal contents of the host animal [7,9]. The choice of a sampling method depends on the research interest, animal distress, simplicity of collection procedure, and ease of transportation to the lab. The sampling phase implies practical benefits and limitations that must be considered before a microbiome-based research plan is chosen [10]. Gut-microbiome studies commonly utilize feces samples to analyze the microbial composition and their metabolites. Collection and transport of feces may be difficult in wildlife because of handling procedures that can cause stress. Rectal or cloacal swabbing to sample intestinal microbiome, without sacrificing the animal, is less preferable to use on small/medium animals due to the risk of injury. Furthermore, the swab-based method in microbiome research is further limited by the small amount of material collected and the difficulty in determining the weight of the sample. The swabbing method procedure requires the development of adjusted DNA extraction protocols, making results often incomparable with standard extraction methods. This sampling method could be also restricted for other omics approaches, such as metabolomics [11]. Therefore, the fecal collection procedure we propose not only might simplify the handling and sampling phases for wild animals, but it could be useful for the processing and archiving phases in all omics research. It provides an almost sterile method in which birds are raised off the ground and do not directly encounter the feces after defecation, proving itself useful in avoiding potential sampling biases, such as contamination and other artifactual biases very common in microbiome research studies [12,13]. Inspired by a useful previously published method for small wild passerine birds [9], we propose an alternative method, used in the Wildlife Rescue Center “Federico II” of Naples (Italy) for animals ranging from 100 gr to 1 kg of body weight and up to 50 cm in height (e.g., common kestrel, *Falco tinnunculus,* or peregrine falcon, *Falco peregrines*). Unlike the previously published method [9], this technique is also more amenable for larger birds. It could be useful to explore the diversity of the microbial landscape, primarily in wild birds but also for other domestic, wild, or exotic bred species, as well as small mammals, for conservation practitioners, wildlife rescue center activities, and veterinary practices.

## 2. Materials and Methods

### 2.1. Fecal Samples Collection Method Design

#### 2.1.1. Materials

**Figure 1:**
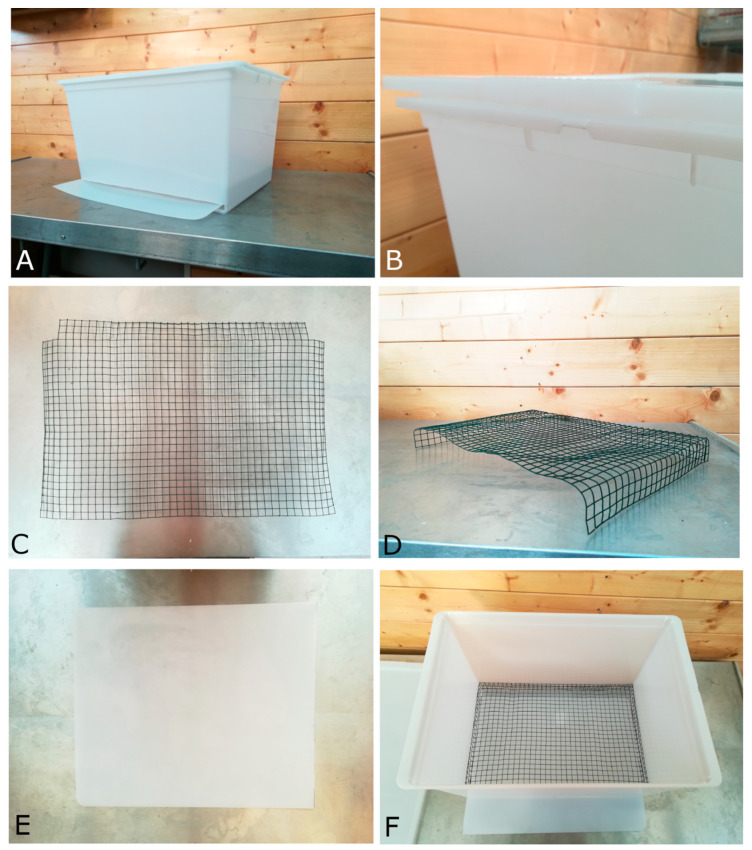
(**A**) The box complete and assembled with the all parts; (**B**) the detailed clip on the lids on the top are useful to close the box safely; (**C**,**D**) The flattened and folded grate that keep the animals raised preventing the contact and contamination of the collecting tray; (**E**) the plastic collecting tray; (**F**) inside view of the box with details of grate and tray.

**Figure 2:**
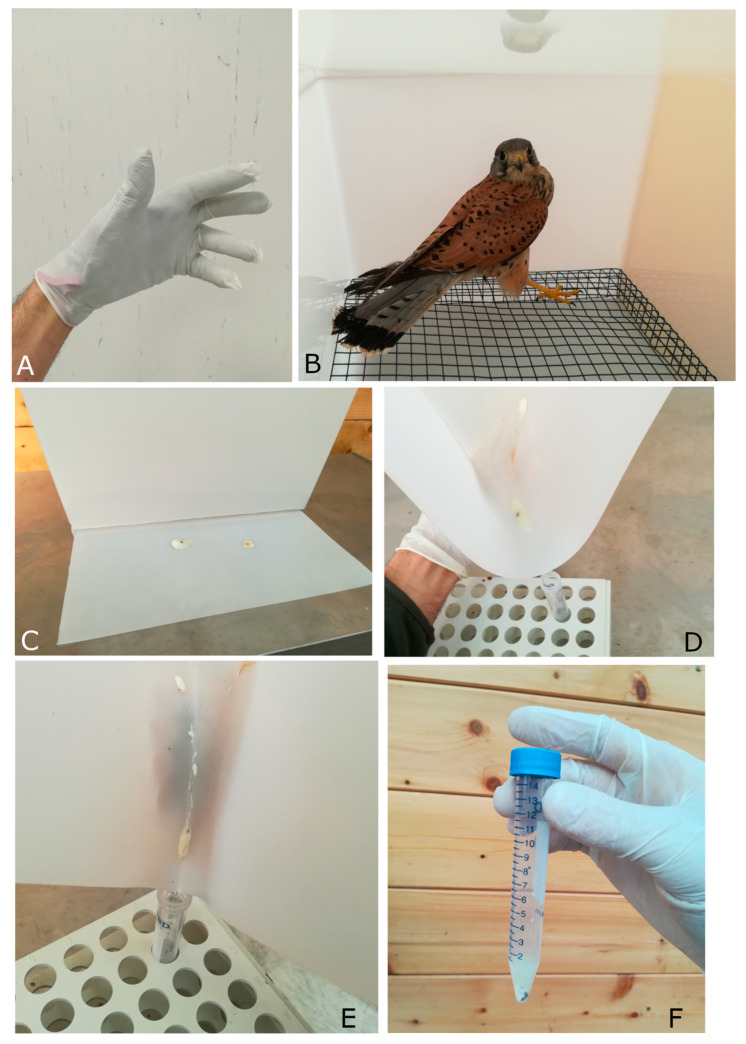
(**A**) The latex gloves cover the leather gloves; (**B**) wild bird (common kestrel, *Falco tinnunculus*) placed in the box; (**C**) fresh feces dropped on the plastic tray; (**D**,**E**) the plastic tray is folded, funnel-like, and the feces are easily removed and collected in the sterile tube; (**F**) the final feces sample obtained ready to be stored or processed.

All materials, listed in Table 1 and illustrated in Figure 1 and Figure 2, are relatively inexpensive (approximately 2500 US Dollars or Euros per kit), and the collection kit is easy to build. The collection kit described in this paper is suitable both for small/medium (100–500 gr) and large birds (500–1000 gr). The kit includes a plastic box (clear and opaque), a hardware cloth, and a plastic tray.

#### 2.1.2. Procedure

First, cut off an approximately 1.5 × 45 cm rectangle shape on one side down of the box. Cut the tray according to the measurements of the bottom of the box—in this case, 45 × 31.5 × 0.3 cm. Then, cut and remove 2 squares (1 square approximately 3.5 cm) from each corner of the cloth (Figure 1C) and fold the outer rows of each side of the cloth to make a 45 × 31.5 × 4.5 cm raised grate (Figure 1D) and place it in the box over the tray (Figure 1F). It is also possible to use this kit for small passerine birds by using a smallest galvanized hardware cloth.

## 3. Results

Individual birds, after recovery and clinical triage [14,15,16], are placed directly in the box (Figure 2B). Wearing latex gloves on the leather gloves (Figure 2A) commonly used for handling of birds of prey, the box is then closed by clips on the lid (Figure 1B) to avoid the escape of the animals. In our experience, birds typically defecate from 5 min to 1 h after arrival. Once the bird has defecated, the tray is removed, folded, and placed with the smallest end in the collection tube (Figure 2D,E). If needed, a sterile swab could be used to move the feces into the tube. To make the fecal collection easier it is also possible to use a pre-sterilized aluminum sheet laid on the tray. Any debris should be removed from the tray before the feces are transferred in the tube. Once feces are collected, the tube is stored as soon as possible at −20 or −80 °C, with or without liquid storage buffers that preserve both RNA (e.g., RNAlater) and DNA (solutions as DMSO/EDTA/saturated sodium chloride, DESS) [9], until DNA extraction. The box, the tray, and the grate are entirely sterilized before and after the collection phase with a 10% spray bleach solution for at least 10 min to reduce the environmental and cross-contaminations [17]. Subsequently the kit is cleaned and dried accurately, with a sterile gauze to prevent the DNA degradation caused by bleach before the introduction of the bird [9].

This collection method was tested for several species of wild birds. In particular, the method was tested for the little owl (*Athene noctua*), tawny owl (*Strix aluco*), Eurasian scops owl (*Otus scops*), Eurasian hobby (*Falco subbuteo*), common kestrel (*Falco tinnunculus*), barn owl (*Tyto alba*), long-eared owl (*Asio otus*), short-eared owl (*Asio flammeus*), Eurasian sparrow hawk (*Accipiter nisus*), common pigeon (*Columba livia*), common little bittern (*Ixobrychus minutes*), Eurasian woodcock (*Scolopax rusticola*), common teal (*Anas crecca*), little grebe (*Tachybaptus ruficollis*), European nightjar (*Caprimulgus europaeus*), black-headed gull *(Chroicocephalus ridibundus*), jack snipe (*Lymnocryptes minimus*), common coot (*Fulica atra*), European jackdaw (*Coloeus monedula*), common moorhen (*Gallinula chloropus*), and peregrine falcon (*Falco peregrines*). The last bird fell into the maximum limit range size for this box. All the tested birds were delivered to the Wildlife Rescue Center “Federico II” of Naples by private citizens, law enforcement officials and/or voluntary associations.

## 4. Discussion

Investigations on the composition and function of the gut microbiome of wild animals are growing. Climate change, land-use change, environmental contamination, new infectious disease, and antimicrobial resistance are some of the most crucial global threats to biodiversity and should indirectly modify the host-associated microbial diversity in wildlife populations [2,18]. Integrated microbiome, metabolomics, and multi-omics analyses reveal interactions between host and microbiota with the goal of obtaining and elaborating on the amount of information from biological samples. These techniques, through an established and correct sample-collecting method, can provide the necessary information that may lead to the surveillance and study of wild populations, to the development of a comprehensive diagnostic and treatment plan for wildlife, and to the discovery of markers and molecules [3,9,19]. As the interest in gut microbiota of wild birds increases, a good sampling method for identification and characterization of microbiome also represents one of the most important steps in an interdisciplinary research view. Through this approach, we might consider the effects, both direct and indirect, of anthropogenic disturbances on wildlife physiology and health. Here, we described a method for collecting fecal samples, in almost sterile conditions, from wild birds ranging from 100 gr to 1 kg of body weight and up to 50 cm in height, that could minimize potential contamination and allow for cross-study comparisons on gut microbiomes. It is also possible to use this kit for small passerine birds after improvement and adaptation of a smaller galvanized hardware cloth. We recommend that all boxes should be easily cleaned and are safe and comfortable for the patients. Equipment necessary for handling should be chosen and based on the patient’s size, behavior, defense tactics, and planned diagnostics. Handling for sampling should be done in close proximity to the box (or caging system in general), when it is possible [20]. Unlike the previously published collecting method for small wild birds [9], our protocol was also designed, built and sized for medium and large wild birds ranging from 100 gr to 1 kg of body weight and up to 50 cm in height. The plastic box is reusable after careful disinfection, using a brush for the grate to avoid cross-contamination between patients. It is more resistant than a paper bag, especially for birds of prey that have sharp claws and a robust beak. In addition, it could be advantageous to better manage the animals after the triage phase. This collecting method could be utilized in other domestic, wild, or exotic bred species, as well as small mammals (i.e., hedgehogs and porcupines). Finally, this collecting box could also be used as an alternative shelter box in case of the overcrowding phenomenon of wildlife rescue centers due to migration and nesting periods of several birds and other wild species during the spring–summer seasons.

## Figures and Tables

**Table 1:** All Materials used for the fecal collecting method with the examples of brands/sellers.

Supplies	Dimensions	Suggested Brands/Sellers
Plastic box 50 L	39.5 × 59.5 × 29 cm	amazon.com
Plastic tray (Desk pad, EVA plastic)	45 × 31.5 × 0.3 cm	ikea.com
Galvanized hardware cloth (vinyl coated)	45 × 31.5 × 4.5 cm; 19 gauge; 1.27 cm (mesh)	amazon.com
Sterile swabs	15.24 cm	Fisherbrand (Fisher Scientific)
Sterile collection tubes (snap or screw top)	5 mL	Thermoscientific (Fisher Scientific)
Bleach	500 mL	Clorox (Amazon.com)
Plastic spray bottle	100 mL	amazon.com
Latex or Nitrile gloves	NA	Fisherbrand (Fisher Scientific)
Sterile gauze	NA	amazon.com
